# Forecasting crew fatigue risk on international flights under different policies in China during the COVID-19 outbreak

**DOI:** 10.3389/fpubh.2022.996664

**Published:** 2022-10-18

**Authors:** SUN Junya, SUN Ruishan

**Affiliations:** College of Safety Science and Engineering, Civil Aviation University of China, Tianjin, China

**Keywords:** policy evaluation, COVID-19, alertness, flight crew, fatigue, risk prediction

## Abstract

To predict the risk of fatigue for flight crews on international flights under the new operating model policy of the civil aviation exemption approach policy during the COVID-19 outbreak, and to provide scientific validation methods and ideas for the exemption approach policy. This paper uses the change in flight crew alertness as a validation indicator, and then constructs an alertness assessment model to predict flight crew fatigue risk based on the SAFTE model theory. Then, the corresponding in-flight rotation plans for the flight is designed according to the exemption approach policy issued by the CAAC, the CCAR-121 part policy and the real operational requirements of the airline, respectively, and finally the simulation results is compared by comparing the pilot alertness and cockpit crew alertness under the exemption approach policy and the CCAR-121 part policy with the flight duration. The results show that the flight crew alertness level for the flight in-flight rotation plan simulation designed under the exemption approach policy is higher or closer to the pilot alertness level for operational flights under the CCAR-121 Part policy. This validates the reasonableness and safety of the exemption approach policy issued by the CAAC to meet the requirements of epidemic prevention and control, and provides scientific support and solutions for fatigue monitoring and management.

## Background

Since the COVID-19 outbreak, the Civil Aviation Administration of China (CAAC) has introduced a number of measures to prevent and control the outbreak. In particular, in order to meet the requirements of passenger and cargo transportation in emergency situations and to effectively protect the health of crew members, as well as to cope with the regular management of the COVID-19 epidemic and to regulate the management of extended crew duty periods and flight time for multiple sets of crews operating on intercontinental routes, Document 2020 No. 53 “Implementation Measures for Exemption of Crew Duty Periods and Flight Time Restrictions during the Epidemic” (hereinafter referred to as the “exemption approach policy”) is formulated and issued (1). The exemption approach policy proposed the operation mode of continuous round-trip flight with multiple crews, which makes the flight crew duty period and flight time exceed the limits of the former “Rules for the Operational Qualification of Carriers of Public Air Transport for Large Aircraft” (China Civil Aviation Regulations-121, CCAR-121 part policy) (2). Table 1 compares the restrictions on flight time for flight crews between the exemption approach policy and the CCAR-121 Part policy (1, 2), and finds that the maximum flight time in the exemption approach policy is 8–13 h more than that of CCAR-121 part policy, and the number of crew members equipped is twice as much as that of CCAR-121 part policy, i.e., the exemption approach policy intends to mitigate crew fatigue measures by increasing the number of flight crews and optimizing rest facilities on board aircraft, which in turn extends it is expected that the exemption approach policy will effectively reduce the risk of crew members contracting epidemics, reduce the workload of the crew and alleviate the risk of crew fatigue, thus ensuring the safe and reliable operation of flights. However, there is a lack of theoretical analysis and scientific validation of the safety and risk of pilot fatigue associated with this mode of operation, which exceeds the limits of the previous regulations and attempts to increase flight time by increasing the number of people.

**Table 1:** Restrictions on the flight time of the crew in the immunization exemption approach policy and CCAR-121 part policy.

**Policy**	**Crew properties**	**Number of crew members/person**	**Flight type**	**Maximum flight duty period/h**	**Maximum flight time limit/h**	**Transit break arrangements**
CCAR-121 part policy	Expansion	3	Passenger / Cargo Classes	16–18	13	Receive a rest period of at least 10 consecutive hours
		4		18–20	17	
Exemption approach policy	Expansion	6	Passenger to cargo/cargo class/separate rest area passenger class	30	26	A ground break of at least 3 consecutive hours and in a rest environment meeting the requirements of a Level 2 rest facility and the break is not counted as part of the flight duty period
			No separate rest area for guest classes	26	21	
		8	Passenger to cargo/cargo class/separate rest area passenger class	35	30	
			No separate rest area for guest classes	26	21	

There are more methods to predict and monitor changes in pilot alertness and fatigue risk, of which biomathematical modeling is a better optional tool among the prediction methods, and it is also a scientific analysis method that is now internationally accepted (3). For example, in 2012, the Fatigue Risk Management System document published by ICAO positioned biomathematical modeling as a viable method for predicting flight crew fatigue risk identification (4). A biomathematical model is a series of mathematical models in the form of a system of equations using physiological parameters related to the organism as input data. It integrates scientific research and flight production planning/scheduling related to fatigue risk, such as human circadian rhythms, sleep, workload and alertness, to better visualize the trend of fatigue during the planned duty period and to predict potential fatigue risk (3, 4). Biomathematical models can therefore assist in the development of optimal scheduling schedules, as well as risk assessment of scheduling schedules and can optimize crew pairings and scheduling costs, in addition to providing assistance in the investigation of safety incidents. Most of the biomathematical models currently used for predictive assessment of fatigue risk have been constructed and developed on the basis of two processes of sleep regulation (5), such as the three-process model of alertness (TPMA) (6), the circadian alertness simulator (CAS) (7), the system for aircrew fatigue evaluation (SAFE) (8), and the sleep/wake predictor (SAFE) (9), fatigue dynamic fatigue audit interDyne (FAID) (10), and Interactive Neurobehavioral Model (INM) (11). In addition, in 2003, Hursh (12) developed a model of sleep, activity, fatigue and task efficiency (SAFTE) based on sleep-activity patterns, circadian rhythms and sleep inertia processes. The model can predict the alertness of the human body at each moment of the day and gives the alertness value for each moment of attention. Therefore, it can be widely used to predict and monitor the fatigue risk of human body in the process of work.

### SAFTE model inputs and outputs

The model inputs are “previous or predicted sleep and activity patterns of the person”; the model outputs are “Modeling circadian oscillators for humans, Calculating the amount of effective sleep in a sleep reservoir based on a person's sleep and activity patterns, and calculate the efficiency of performing tasks based on the above oscillations and sleep reservoirs based on the sleep/wake data, etc.” Note in particular that the predicted outcome of alertness (effectiveness) is usually expressed as a change in cognitive validity based on a comparison of baseline levels in percentage terms (12). Therefore, the alertness (effectiveness) measures calculated by SAFTE are expressed as percentage. Based on the characteristics of the biomathematical model that “comparing scores is better than complying with thresholds” (13, 14), The SAFTE model therefore also does not provide an acceptable level of alertness. That is, the predictions are derived by comparing the percentage values of alertness at two points in time (12). However, the SAFTE model also reports that studies of total sleep deprivation show that cognitive capacity is depleted at a rate of approximately 25% per day, such an extreme level of alertness (12).

### SAFTE model calculation measures for alertness (effectiveness)

The circadian process influences both performance modulation and sleep regulation. The performance modulation, including factors are sleep inertia, circadian rhythm, performance and effectiveness, the performance modulation depends on the circadian process, sleep inertia, and the current balance of the sleep reservoir. The sleep regulation, including factors are sleep quality, sleep intensity, sleep accumulation, sleep debt and fragmentation (namely awakenings during periods of sleep), the sleep regulation depends on the hour of asleep and awake, the circadian process and sleep debt. Mathematical modeling can be used to simulate the physiological processes mentioned above, this can be implemented on software such as matlab for general purpose digital computers (12, 15, 16).

This paper presents numerical simulation predictions of flight crew and crew alertness in the cockpit under both exemption approach policy and CCAR-121 part policy restrictions. By comparing the exemption approach policy with the flight fatigue risk prediction results of CCAR-121 part policy, the problem of assessing and monitoring the fatigue risk of crew members at various moments during duty and flight is solved, and the problem of measuring the fatigue risk of pilots is also solved, which is of great significance to the pre-intervention of flight fatigue of crew members before duty, the monitoring of changes in the cognitive ability of crew members in the cockpit and the safeguarding of flight safety on routes.

## Modeling of a flight crew alertness assessment model based on SAFTE

One potential application of the SAFTE model is that the effectiveness of a crew's work during the day can be assessed based on past or future sleep patterns (12). We therefore construct an alertness assessment model for the predictive assessment of alertness during a single flight for exempt flight crews.

### Performance rhythmic processes

The circadian oscillators (12, 15, 16) are represented by the following equation:


(1) $$\begin{array}{l} c=\cos\left(2\pi \left(T-p\right)/24\right)+\beta \cos\left(4\pi \left(T-p-p^{\prime }\right)/24\right) \end{array}$$

Where, *T i*s the time of day, *p* is the cosine phase with a period of 24 h, *p*'is the cosine phase with a period of 12 h, β is the cosine amplitude with a period of 12 h.

The performance rhythm (12, 17) is represented by the following equation:


(2) $$\begin{array}{l} C=a_{p}\times c \end{array}$$

Where, *c* is the circadian oscillators; *a*_p_ is the amplitude of the alertness rhythm (12), is represented by the following equation:


(3) $$\begin{array}{l} a_{p}=a_{1}+a_{2}\left(R_{c}-R_{t}\right)/R_{c} \end{array}$$

Where, *a*_1_ is a constant alertness rhythm amplitude factor, *a*_2_ is a variable alertness rhythm amplitude factor, *R*_c_ is the total capacity of the sleep reservoir, *R*_t_ is the capacity of the sleep reservoir at time *t*.

### Sleep-wake homeostatic processes

Described in terms of a sleep “reservoir” (12), the sleep-wake homeostatic process can be described as an equilibrium *R*_*t*_ process in the current sleep reservoir, regulated by sleep accumulation and wakefulness consumption, with the following expressions:


(4) $$R_{t}=\{\begin{array}{c} R_{t-1}+S,\text{Sleeping period}\\ R_{t-1}-P,\text{Awakening period} \end{array}$$

Where, *R*_t_ is the capacity of the sleep reservoir at time *t*, *R*_t − 1_ is the capacity of the sleep reservoir at moment *t-1, S* is sleep accumulation, *P* is alertness consuming.

Equation (4) relates to the sleep accumulation *S* in the sleep stage, consisting of sleep intensity and sleep debt, which is expressed as follows (12):


(5) $$\begin{array}{l} S=S_{I}\times t^{\prime \prime } \end{array}$$

(6) $$\begin{array}{l} S_{I}=S_{P}\times S_{D} \end{array}$$

(7) $$\begin{array}{l} S_{P}=m-\left(\;a_{s}\times c\right) \end{array}$$

(8) $$\begin{array}{l} S_{D}=f\left(R_{c}-R_{t}\right) \end{array}$$

Where, *S*_*I*_ is the sleep intensity, *t*″ is the sleep time, *S*_*P*_is the sleep tendency, *S*_*D*_ is the sleep Debt, *m* is the sleep propensity value, *a*_s_ is the sleep tendency amplitude, *c* is the function of circadian rhythm, *f* is the feedback amplitude, *R*_c_ is the total capacity of the sleep reservoir, *R*_t_ is the capacity of the sleep reservoir at time *t*.

Equation (4) relates to the alertness consumption *P* during the awakening phase and consists of the product of the alertness consumption rate and the working time, which is expressed as follows (12):


(9) $$\begin{array}{l} P=K\times t^{\prime } \end{array}$$

Where, *K* is the rate of alertness consumption, *t*′ is the working hours.

### Sleep inertia processes

The third factor, sleep inertia *I*, is influenced by the moment of awakening and the intensity of sleep and is expressed as follows (6, 12):


(10) $$\begin{array}{l} I=\;I_{\text{max}}\times e^{-\left(t_{a}/S_{I}\times i\right)} \end{array}$$

Where, *I*_max_is the maximum sleep inertia value, *t*_a_ is the moment of awakening, *S*_*I*_is the leep intensity, *i* is the time constant of inertia 2 h after awakening.

From the above equation, the model outputs the human alertness *E* at moment *t* of the day, which is expressed as follows (12):


(11) $$\begin{array}{l} E=100\left(R_{t}/R_{c}\right)+C+I \end{array}$$

Where, *R*_c_ is the total capacity of the sleep reservoir, *R*_t_ is the capacity of the sleep reservoir at time *t*; *C* is alert rhythms; *I* is the sleep inertia.

In addition, Table 2 gives the default values for the parametric variables used in the model.

**Table 2:** Default values of relevant parameter variables in the cognitive effective competence assessment model.

**Parameters**	**Explanation**	**Default value**
*p*	Cosine phase with a period of 24 h	18
*p*'	Cosine phase with a period of 12 h	3 h ahead of p (is p+3)
β	Cosine amplitude with a period of 12 h	0.5
*m*	Sleep propensity values	0
*a*_s_	Sleep tendency amplitude	0.55
*a*_1_	Constant alertness rhythm amplitude factor	0.07
*a*_2_	Variable alertness rhythm amplitude factor	0.05
*R*_c_	Total sleep reservoir capacity	2,880 - Units required for 4 consecutive days without sleep
*f*	Feedback amplitude	0.0026243
*i*	Time constant of inertia 2 h after awakening	0.04
*I*_max_	Maximum sleep inertia after awakening	0.05
*K*	alertness consumption rate	0.5/min
*t*	A particular calculation time	-
*t'*	Time interval when working	1 min
*t″*	Time interval when sleeping	1 min

The above constructed flight crew alertness assessment model based on SAFTE was developed in code on Matlab R2020b software. In addition, the time involved in the model and the code is determined by the plans of the rotation during the in-flight, the following will design the flight crew work rotation plans during the in-flight in accordance with the exemption policy and the CCAR-121 policy.

## Design of in-flight rotation plans for flights under both exemption approach policy schemes and CCAR-121 part policy

The in-flight rotation plan for flights under both the exemption approach and CCAR-121 part policies are designed according to the policy restrictions of the Bureau and the operational requirements of an airline mentioned above. All times in the flight are in Beijing time and the flight crew consists of 2 pilots each. The specific in-flight rotation schedule is shown in Tables 3–6.

**Table 3:** Flight work plan for a particular flight under exempted approach operation (3 sets of expansion crews).

**Time of day Crew**	**Crew A**	**Crew B**	**Crew C**
10:00–12:00	Flying	Resting	Resting
12:00–16:00	Sleeping	Flying	Sleeping
16:00–20:00	Resting	Resting	Flying
20:00–22:00	Flying	Sleeping	Sleeping
22:00–1:00 at destination	Sleeping
1:00–3:00	Flying	Sleeping	Sleeping
3:00–7:00	Sleeping	Flying	Sleeping
7:00–11:00	Sleeping	Sleeping	Flying
11:00–13:00	Flying	Resting	Resting

A, B, and C are all 2 pilots (main and co-pilot).

**Table 4:** Flight work plan for a particular flight under CCAR-121 part policy department operations (3 expansion crews).

**Time of day crew**	**Pilot A**	**Pilot B**	**Pilot C**
10:00–13:00	Flying	Resting	Flying
13:00–16:00	Flying	Flying	Sleeping
16:00–19:00	Flying	Flying	Resting
19:00–22:00	Sleeping	Flying	Flying
22:00–5:00 at destination	Sleeping	Sleeping	Sleeping
5:00–8:00 at destination	Resting	Sleeping	Sleeping
8:00–11:00	Flying	Resting	Flying
11:00–14:00	Sleeping	Flying	Flying
14:00–17:00	Resting	Flying	Flying
17:00–20:00	Flying	Flying	Sleeping

**Table 5:** Flight work plan for a particular flight under exempted approach operations (4 sets of expansion crews).

**Time of day crew**	**Crew D**	**Crew E**	**Crew F**	**Crew G**
20:00–23:00	Flying	Resting	Resting	Resting
23:00–3:30	Sleeping	Flying	Sleeping	Sleeping
3:30–8:00	Sleeping	Sleeping	Flying	Sleeping
8:00–11:00	Resting	Sleeping	Sleeping	Flying
11:00–14:00 at destination	Sleeping
14:00–17:00	Flying	Resting	Resting	Resting
17:00–21:30	Resting	Flying	Sleeping	Sleeping
21:30–2:00	Sleeping	Sleeping	Flying	Sleeping
2:00–5:00	Sleeping	Sleeping	Sleeping	Flying

D, E, F, and G are all 2 pilots (main and co-pilot).

**Table 6:** Flight work plan for a particular flight under CCAR-121 part policy section operations (4 pilots expansion crews).

**Time of day crew**	**Pilot D**	**Pilot E**	**Pilot F**	**Pilot G**
20:00–0:30	Flying	Flying	Sleeping	Sleeping
0:30–8:00	Sleeping	Sleeping	Flying	Flying
8:00–11:00	Flying	Flying	Resting	Resting
11:00–21:00 at destination	Sleeping
21:00–1:30	Sleeping	Sleeping	Flying	Flying
1:30–9:00	Flying	Flying	Sleeping	Sleeping
9:00–12:00	Resting	Resting	Flying	Flying

## Results

Combining the constructed flight crew alertness assessment model and the designed in-flight rotation plan, Matlab R2020b software is applied to carry out model simulation calculations, and the results are as follows.

**Figure 1:**
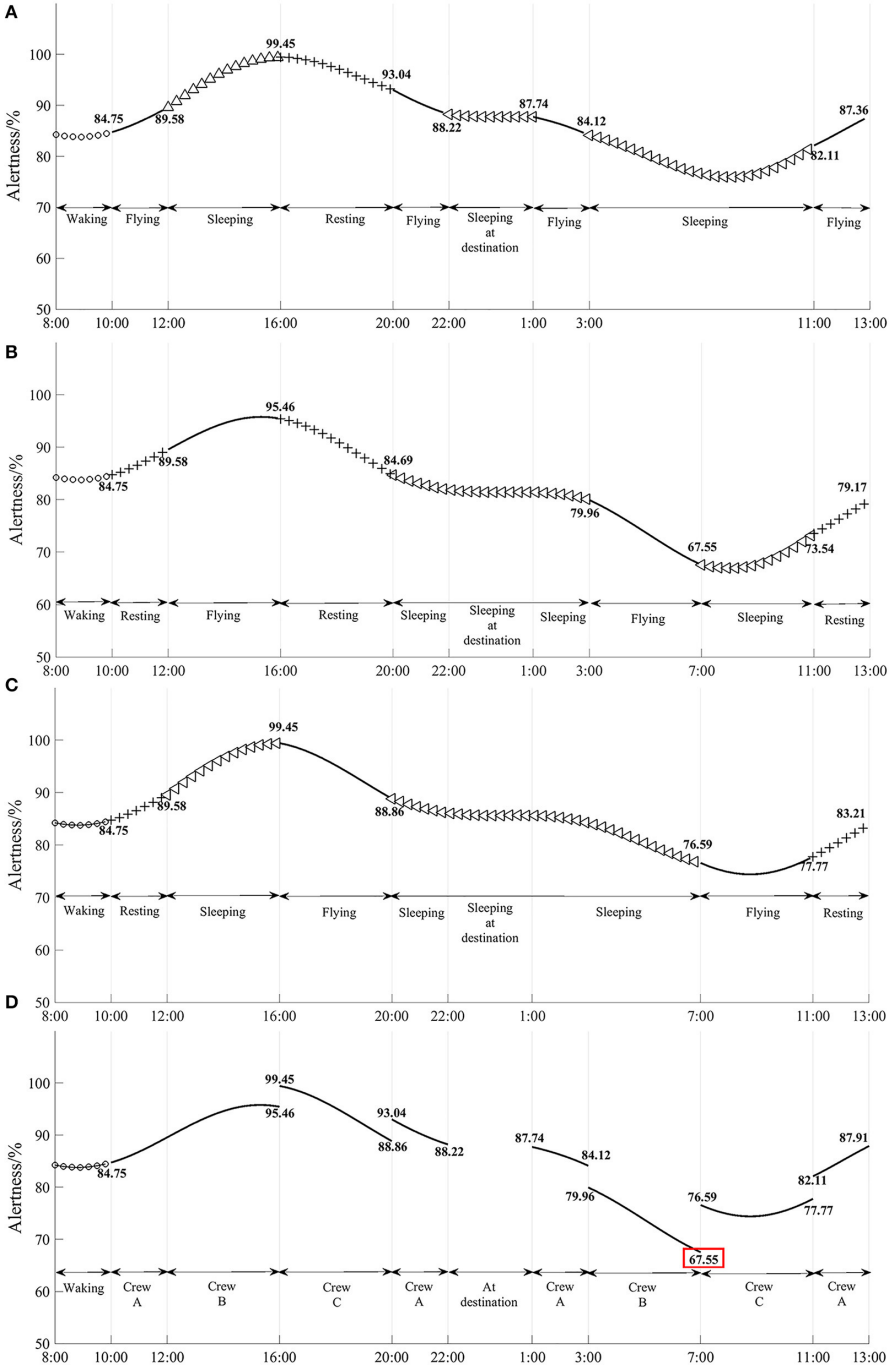
**(A–D)** Variation in the alertness of each flight crew and its cockpit crew with flight hour for flights subject to the exemption approach policy (3 sets of crews).

Figure 1A shows the model simulation results of flight crew A's alertness with flight time, it is found that flight crew A's alertness remained above 82.11% during the flight, and the maximum change in alertness before and after shift work is 5.25%; Figure 1B shows the model simulation results of flight crew B's alertness with flight time, it is found that flight crew B's alertness is at its lowest point at the end of the return flight shift, at 67.55%, and the maximum change in alertness before and after shift work is 12.41%. The lowest point of alertness is 67.55%, and the maximum change in alertness before and after the shift is 12.41%. Figure 1C shows the results of the model simulation of the change in alertness of flight crew C with flight duration, and it is found that the lowest point of alertness of flight crew C is 74.41% during the return flight shift, but the lowest point occurred during the flight shift, and the maximum change in alertness before and after the flight shift is 10.59%. The lowest point of alertness is 67.55% at the end of Flight Crew B's return shift, and the lowest point of alertness is found during the cruise phase of the return flight.

**Figure 2:**
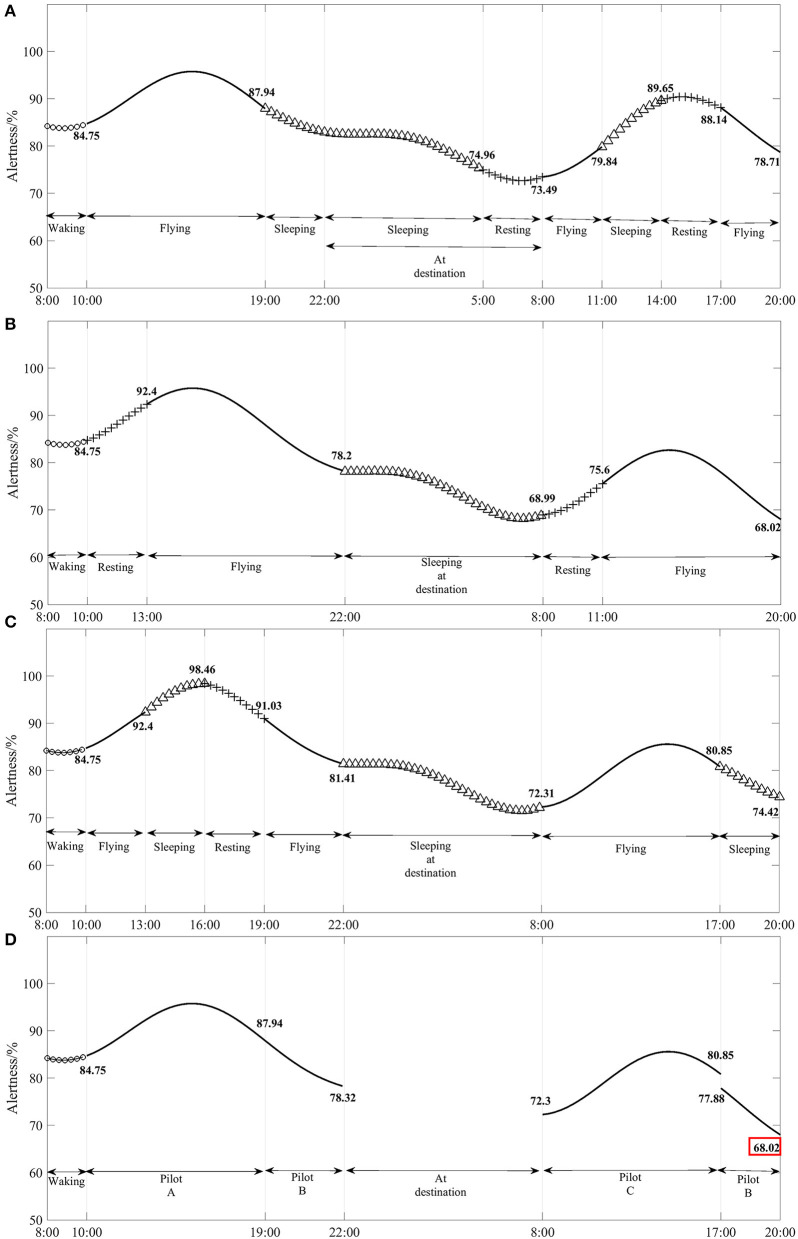
**(A–D)** Variation in flight crew and their cockpit crew alertness with flight hour for flights under CCAR-121 part policy restrictions (3 pilots).

Figure 2A shows the model simulation results of Pilot A's alertness with flight duration, and it is found that Pilot A's alertness remained above 73.49% during the flight, with a maximum change of 9.43% before and after shift work. The lowest point of alertness is 68.02%, and the maximum change in alertness before and after the flight shift is 14.2%. Figure 2C shows the model simulation results of Pilot C's alertness as a function of flight time. It is found that Pilot C's alertness remained above 72.31% throughout the flight, with a maximum change of 9.62% before and after the shift work. The lowest point of alertness is 68.02% when Pilot B landed on the return leg.

**Figure 3:**
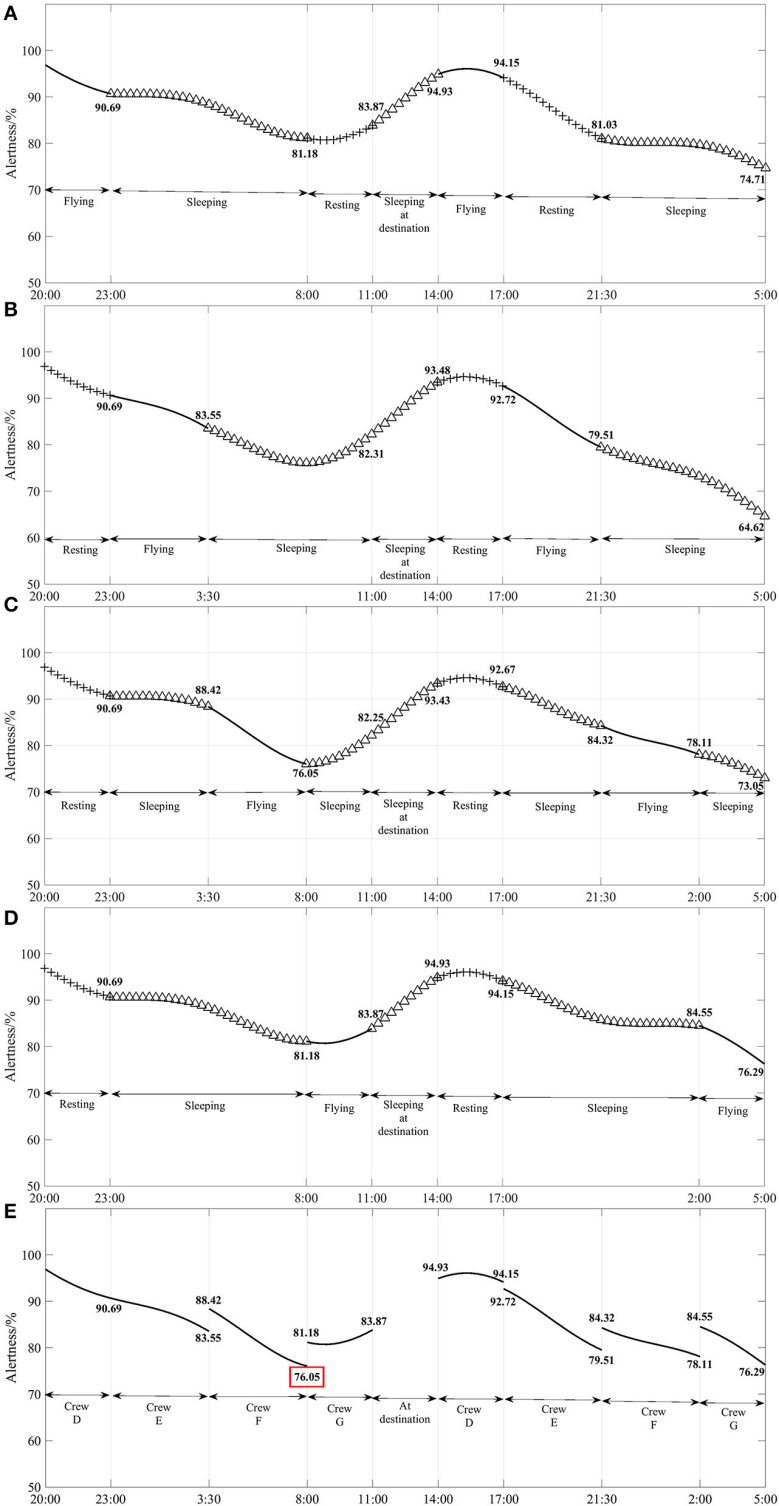
**(A–E)** Variation in flight crew alertness by flight crew and their cockpit crew with flight hour for flights subject to the exemption approach policy (4 sets of crews).

Figure 3A shows the model simulation results of the change in alertness of Flight Crew D with flight duration. Figure 3B shows the model simulation results of Flight Crew E's alertness as a function of flight time, and it is found that Flight Crew E's alertness remained at 79.51% throughout the flight and shift work phases, and the maximum change in alertness before and after shift work is 13.21%. Figure 3C shows the model simulation results of the change in alertness of Flight Crew F with flight time, and it is found that the lowest point of alertness of Flight Crew F is 76.05% at the end of the departure shift, and the maximum change in alertness before and after the shift is 12.37%. The lowest point of alertness is 76.29% during landing, and the maximum change in alertness before and after shift work is 8.26%. Figure 3E shows the results of the model simulation of the change in crew alertness in the cockpit with flight duration. The lowest point of alertness throughout the flight occurred at 76.05% at the end of the flight crew G departure shift.

**Figure 4:**
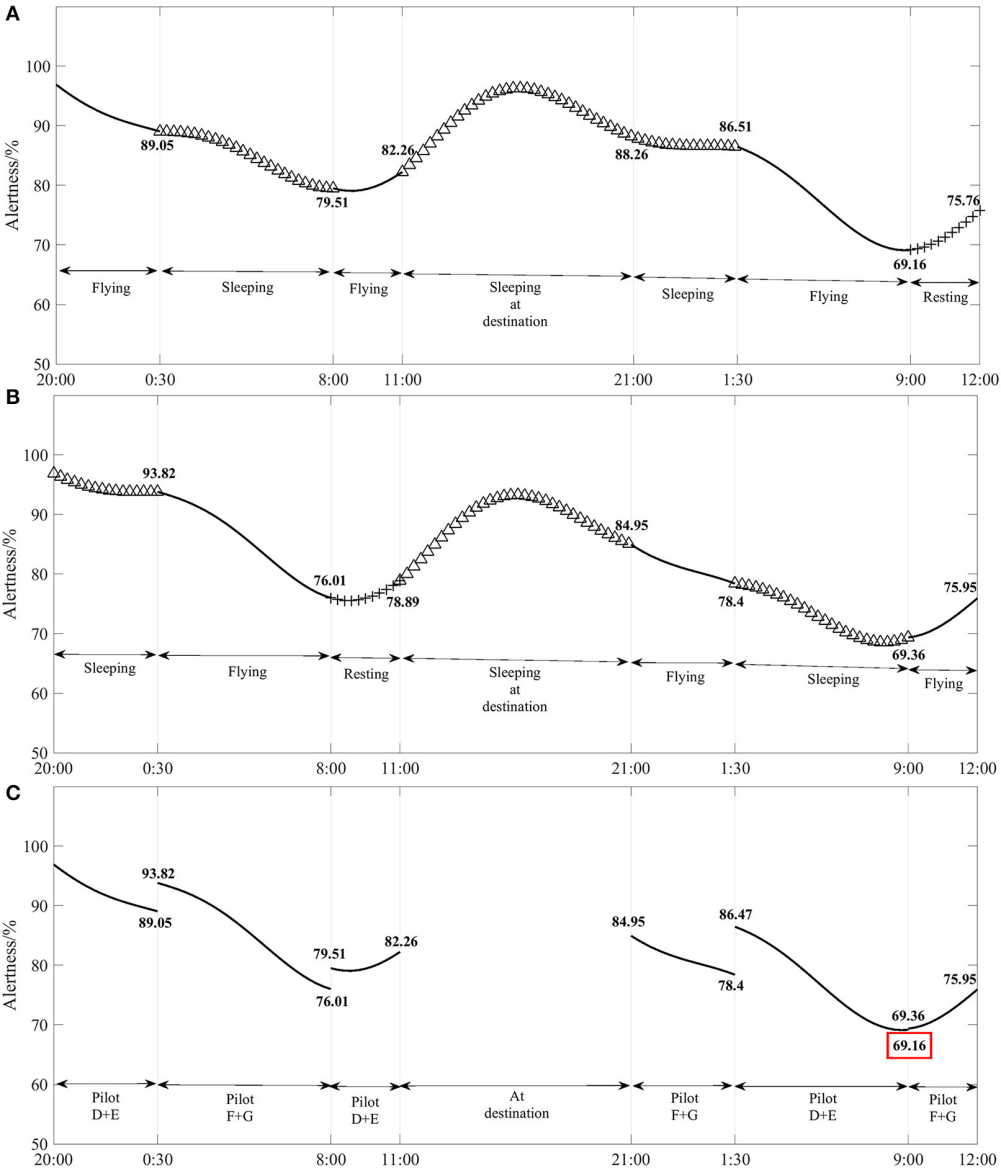
**(A–C)** Variation in the alertness of each flight crew and their cockpit crew with flight duration for flights under CCAR-121 part policy restrictions (4 pilots).

Figure 4A shows the results of the model simulation of the change in alertness of pilots D and E (pilots D and E are in the same flight crew and have cockpit flying duties together). (Pilots F and G are in the same flight crew) and found that Pilots F and G had the lowest point of alertness at the beginning of their return landing flight duty, 69.36%, and the maximum change in alertness before and after their shift work is 17.81%. Figure 4C shows the results of the model simulation of the change in cockpit crew alertness with flight time. The lowest point of alertness throughout the flight occurred during the return pilot shift handover, with a minimum of 69.16%. The change in alertness shows that there is a relatively large change in alertness for all pilots.

## Discussion

As can be seen from Table 1, the maximum duty period hours and flight time hours in the exemption approach policy is close to twice that of CCAR-121 part policy, so the exemption approach policy faces a number of personnel fatigue issues for extra long hours of duty and flight, such as long working hours which obviously lead to sleep deprivation and circadian rhythm factors which complicate all-weather work (18). Goode (19) found that the probability of commercial aviation accidents increased significantly with increasing duty time, with 20% of US commercial aviation accidents appearing to occur at 10 h or more. Thus, in addition to circadian rhythm disturbances and acute or cumulative sleep deprivation, prolonged periods of continuous wakefulness can also significantly increase pilot fatigue.

In addition, the exemption approach policy is faced with an extraordinarily long low workload cruise phase of flight, and Cabon et al. (20) have shown that long-haul pilots are particularly vulnerable to alertness failures during low workload periods. Furthermore, it has been established that these lapses can occur with two crew members at the same time (an obvious safety issue). Wright N and McGown A (21) similarly found that pilot microsleeps occurred most frequently during the cruise portion of long-haul flights (mid to late in the flight), were more than nine times more likely to occur compared to night flights, and found that this spontaneous microsleep increased with flight duration. Therefore, attention should be paid to alertness lapses during cruising.

At the same time, the number of matches in the exemption approach policy is twice as large as in CCAR-121 part policy, so there is also the issue of “rest and rotation of personnel on board” in the exemption approach policy. For long-haul flights, especially when flying with extended crews, rest and rotation is inevitable to avoid flight time constraints and to relieve fatigue. Most commercial aircraft designed for long-haul flights, such as the Boeing 747-400 and Airbus A340, are equipped with rest facilities that can be used by extended crews (22). Although sleep in rest facilities is reportedly less rejuvenating than sleeping in a hotel or at home (23), these opportunities to sleep using in-flight rest facilities are more beneficial than trying to sleep in a chair, while any opportunity to sleep is better than being constantly awake (24). So, as per the 3 types of in-flight rest facilities specified in CCAR-121 part policy, the exemption approach policy specify that in-flight rest facilities should be no less than Class 2 standard.

The exemption approach policy is a temporary policy developed in response to the COVID-19 epidemic and therefore the safety of this method of management, which is based on an increase in numbers and therefore in flight time, is not yet scientifically proven. In fact, many industry organizations and regulatory bodies have attempted to manage fatigue by setting working hours, i.e., these rules usually specify maximum working hours and minimum rest periods, but they take little or no account of the physiological determinants of fatigue (e.g., the impact of circadian rhythms). However, to some extent, the lack of simple, reliable and effective fatigue management tools has forced the use of time-based rule-based approaches (10, 25, 26). In June 2002, a workshop on fatigue and performance modeling is held in Seattle, Washington, to which seven research groups from Australia, Europe and the USA were invited to participate, each developing a model for estimating fatigue associated with shift work (27). During the workshop, different models were used to predict fatigue in different scenarios and a comparison of the results showed that the models each had strengths and weaknesses (27–29). One of the main applications of the SAFTE model, developed by Hursh et al. (12), which aims to model the underlying physiological systems that contribute to personnel cognitive decline and to estimate the decline in alertness due to human fatigue, is to help managers develop work plans by using work plan information to estimate cognitive performance such as personnel fatigue and alertness. In addition, the SAFTE model can distinguish between “in-flight” and “non-in-flight” events and can estimate alertness and sleep reservoir separately for each event (12, 17). Therefore, the SAFTE model is used to develop the flight crew alertness assessment model.

Based on the characteristics of the biomathematical model that “comparing scores is better than complying with thresholds” (13, 14), and the fact that the exemption approach policy is a temporary deviation from the duty period and flight time limits for crew members based on CCAR-121 part policy, it is necessary to use the existing flight time limits in CCAR-121 part policy as a benchmark for comparison, and to analyse the feasibility and scientific validity of the extended flight time provisions in the exemption approach policy.

Based on the model simulation results in Figures 1, 2, it is found that the minimum alertness throughout the flight under the exemption approach policy is only 0.47% less than that under CCAR-121 part policy, and that the alertness of pilots on shift duty under both policies is above 67.55%. Therefore, the three sets of flight crew alertness for the exemption approach policy remained at a similar level to CCAR-121 part policy.

Based on the results of the model simulation calculations in Figures 3, 4, it is found that the minimum alertness throughout the flight under the exemption approach policy is 6.89% higher than that under CCAR-121 part policy, and that the alertness of pilots flying on shift duty under both policies is above 69.16%. Therefore, the four sets of flight crew alertness levels for the exemption approach policy is higher than those of CCAR-121 part policy.

In summary, the results of the flight crew alertness assessment model based on the SAFTE model and the above-mentioned flight rotation plan designed in accordance with the CAAC regulations and airline requirements have verified that the overall flight crew alertness of the “3/4 set” flights operating under the exemption approach policy is higher or closer to that of the “3/4” flights under the CCAR-121 part policy. The overall level of pilot fatigue risk under the exemption approach policy is lower or similar to the level of fatigue risk under CCAR-121 part policy, thus validating the feasibility of the exemption approach policy and providing a solution for airlines to predict crew fatigue risk under the exemption approach policy.

## Conclusion

In this paper, a pilot alertness assessment model is constructed to simulate flight schedules under the exemption approach policy and the CCAR-121 Part policy, and the following conclusions are obtained: the above simulation results and analysis, as well as the comparison of flight crew alertness between the two policies, verified that the fatigue and mental state of crew members in the cockpit under the exemption approach policy is similar to and better than that under CCAR-121 part policy overall. In addition, using the parameters assigned in this paper, the study verifies the feasibility and scientific validity of the exemption approach policy and the model provides theoretical support and solutions for airlines' flight planning and crew shift.

## Data availability statement

The original contributions presented in the study are included in the article/supplementary material, further inquiries can be directed to the corresponding author.

## Author contributions

SJ conceived and designed the manuscript and wrote the article. SR edited and revised the article. Both authors contributed to the article and approved the submitted version.

## Funding

This research project was supported by the National Natural Science Foundation of China (52272356) and the Scientific Research Program of Tianjin Education Commission (2020KJ029).

## Conflict of interest

The authors declare that the research was conducted in the absence of any commercial or financial relationships that could be construed as a potential conflict of interest.

## Publisher's note

All claims expressed in this article are solely those of the authors and do not necessarily represent those of their affiliated organizations, or those of the publisher, the editors and the reviewers. Any product that may be evaluated in this article, or claim that may be made by its manufacturer, is not guaranteed or endorsed by the publisher.
